# CCL19 suppresses angiogenesis through promoting miR-206 and inhibiting Met/ERK/Elk-1/HIF-1α/VEGF-A pathway in colorectal cancer

**DOI:** 10.1038/s41419-018-1010-2

**Published:** 2018-09-24

**Authors:** Zhuoqing Xu, Congcong Zhu, Chun Chen, Yaping Zong, Hao Feng, Di Liu, Wenqing Feng, Jingkun Zhao, Aiguo Lu

**Affiliations:** 1 Shanghai Minimally Invasive Surgery Center, Ruijin Hospital, Shanghai Jiaotong University School of Medicine, Shanghai, PR China; 2 Shanghai Institute of Digestive Surgery, Shanghai, PR China

## Abstract

The mechanisms underlying the role of chemokines in tumor angiogenesis is still not fully understood. In this study, we detected the influence of CCL19 on colorectal cancer (CRC) angiogenesis. The expression of CCL19 and CD31 in CRC tissues were detected by immunohistochemistry. Human CRC cell lines SW1116 and SW620 stably transfected with CCL19 lentivirus and CCL19 shRNA, and HUVEC stably transfected with CCR7 shRNA were used in our study. Our study showed that CCL19 was significantly low-expressed in CRC tissues and positively related to highly tumor microvessel density. In vitro, we observed that CCL19 high-expressed SW1116 supernatant was able to inhibit proliferation, migration, and sprouting responses of HUVEC, whereas CCL19 low-expressed SW620 supernatant can promote HUVEC angiogenesis. Additionally, we further demonstrated that these functions maybe achieved through promoting miR-206 thus inhibiting Met/ERK/Elk-1/HIF-1α/VEGF-A pathway in a CCR7-dependent manner. Mice angiogenesis model also confirmed that elevated expression of CCL19 inhibit the angiogenesis of CRC in vivo. In summary, our results supported that CCL19 can inhibit CRC angiogenesis through promoting miR-206 thus inhibiting Met/ERK/Elk-1/HIF-1α/VEGF-A pathway. This may be a novel therapeutic option for anti-vascular treatment in CRC.

## Introduction

Colorectal cancer (CRC) is one of the most common malignant tumors of the digestive system, with morbidity and mortality ranking third in the world^1^. Although many advances have been made in the diagnosis and treatment of CRC, the CRC-related mortality rate remains high^2^. Tumorigenesis, tumor development, and metastasis are a complex and multi-step processes where angiogenesis plays an important role^3^. Despite a increasing number of proteins and signaling pathways have been found to be closely associated with tumor angiogenesis^4^, the role of chemokines in the tumor microenvironment that promote CRC angiogenesis remains unknown.

Chemokines belong to a superfamily that consists of small proteins, which are able to bind to G-protein-coupled receptors that activate downstream accesses and functions^5^. Chemokines and their receptors widely participate in tumorigenesis, metastasis, and angiogenesis^3,6^. Our previous studies revealed that CXCL5, CCR4, and CCR6 are overexpressed in CRC tissues compared with normal tissues, and elevated expression of these factors could promote cancer metastasis and angiogenesis^7–9^. Chemokine CC ligand 19 (CCL19), which is also named as macrophage inflammatory protein 3-beta (MIP-3b), mediates various cellular behaviors by binding to CCR7^10^. Recent articles have indicated that the CCL19/CCR7 axis can promote tumor progression^11,12^. Nevertheless, some other studies indicated that CCL19 can modulate anti-tumor responses in lung cancer and ovarian cancer^13,14^. Similarly, our previous studies demonstrated that CCL19 inhibited tumorigenesis, metastasis and angiogenesis, and the expression of CCL19 was associated with the prognosis of CRC patients^15,16^. However, the potential function of CCL19 expressed in CRC remains to be elucidated.

In this study, we further investigated the mechanisms and signal pathways of CCL19 suppress CRC angiogenesis based on the results of our previous work. We first examined the expression of CCL19 in CRC tissues and found that CCL19 was low-expressed in CRC tissues compared with normal tissues. In addition, we detected the association between CCL19 expression and tumor microvessel density (MVD) of CRC tissues, and the results showed that CCL19 levels were negatively correlated with angiogenesis. Moreover, we also studied the function of CCL19 on angiogenesis in vitro and in vivo. Our study revealed that CCL19 was able to suppresses angiogenesis in CRC through promoting miR-206 thus inhibiting Met/ERK/Elk-1/ HIF-1α/VEGF-A pathway in a CCR7-dependent pattern. Our study confirmed that CCL19 low-expressed in CRC is able to promote tumor angiogenesis, indicating that CCL19 may be a promising therapeutic target in CRC anti-angiogenic treatment.

## Results

### CCL19 is low-expressed in CRC tissues and associated with tumor angiogenesis

**Fig. 1:**
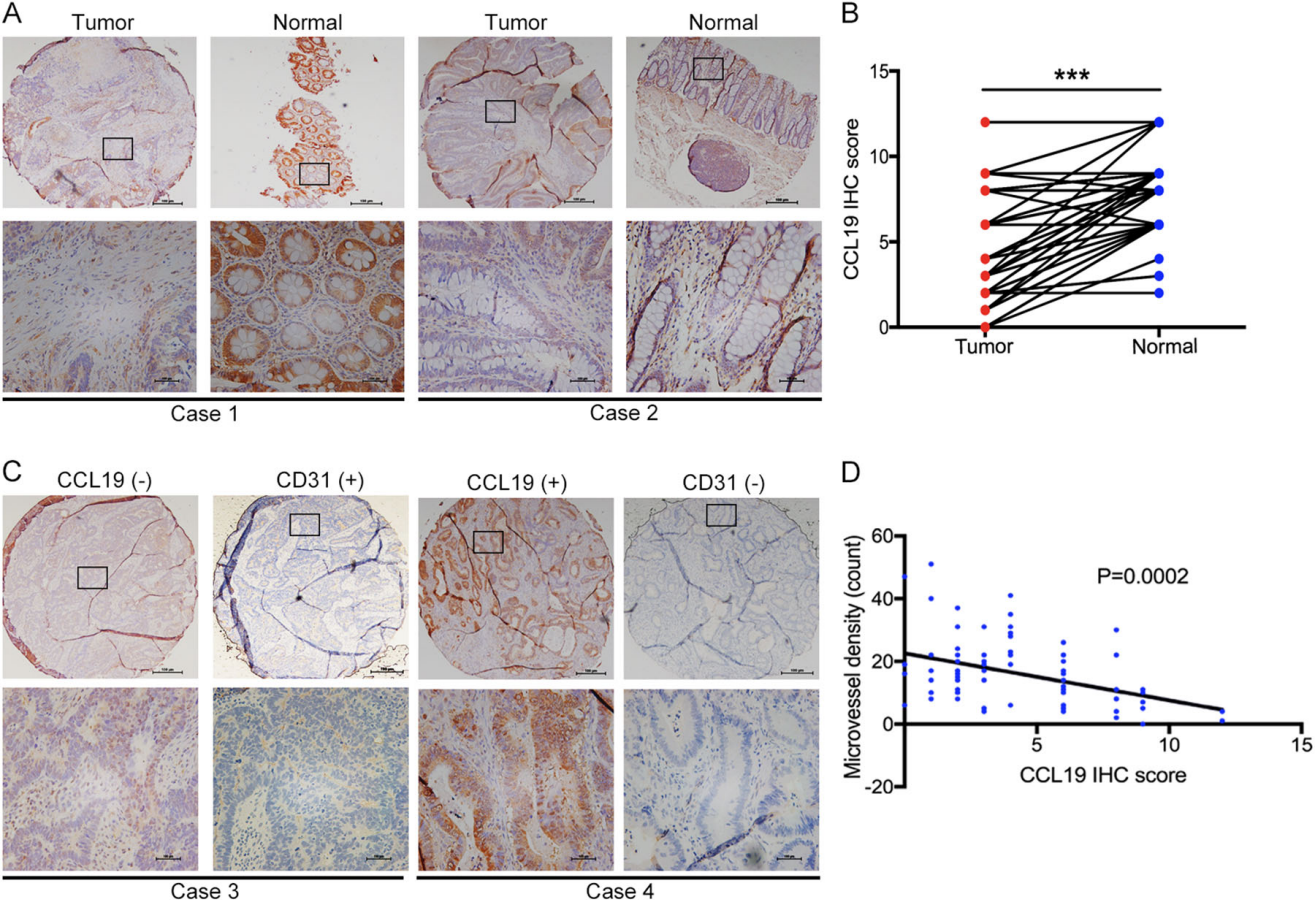
CCL19 is low-expressed in CRC tissue and negatively correlated with tumor angiogenesis. **a** CCL19 expression level in tumor tissues and the paired normal tissues was evaluated by immunohistochemical staining with tissue microarray (scale: 200 μm). **b** CCL19 is lowly expressed in cancer tissues compared with normal tissues in tissue microarray. **c** CCL19 expression level and MVD (CD31-positive cell) in tumor tissues (scale: 200 μm). **d** Expression correlation of CCL19 and MVD was analyzed in 78 CRC patients (*P* = 0.0002). Data represent the mean ± SD. *P < 0.05, **P < 0.01, ***P < 0.001

Immunohistochemistry assay was conducted to detect the expression of CCL19 in 78 pairs of CRC tumor and adjacent normal tissues. As shown in Fig. 1a, b, the CCL19 level was significantly down-regulated in CRC tissues compared with peritumoral normal tissues (*P* < 0.0001). We selected a staining score of 4.3 as the cut-off value using X-tile software (Supplementary figure 1A). In this cohort, CCL19 expression was down-regulated in ~61.5% (48/78) of the paired tissues (Table 1). To explore the clinicopathological significance of CCL19 in CRC, we analyzed the correlation between CCL19 expression and CRC patients’ clinical and pathologic materials in the TMA cohort (Table 1). The analysis revealed that the low expression of CCL19 was significantly correlated with Dukes’ stage (*P* = 0.003). In addition, we analyzed the association between CCL19 expression and tumor angiogenesis of CRC patients. Correlation analysis demonstrated that low-expressed CCL19 in CRC tissues was significantly associated with high MVD (*P* = 0.0002, Fig. 1c, d).

**Table 1:** Correlations between CCL19 expression and clinical characteristics in CRC patients

Clinicopathologic parameters	Case (*n* = 78)	CCL19 expression		Statistical value	*P*- value
		Positive	Negative		
Total	78	30	48		
Gender				0.680	0.410
Male	41	14	27		
Female	37	16	21		
Age				3.759	0.053
≥65	31	16	15		
<65	47	14	33		
Histology				1.95	0.377
Tubular	66	24	42		
Mucinous	11	5	6		
Papillary	1	1	0		
Tumor location				3.385	0.496
Right hemicolon	25	13	12		
Transverse colon	1	0	1		
Left hemicolon	6	2	4		
Sigmoid + Rectum	46	15	31		
Tumor size				0.329	0.566
≥5 cm	37	13	24		
<5 cm	41	17	24		
Dukes stage				17.8	0.0032
I	15	10	5		
II	33	17	16		
III	24	3	21		
IV	6	2	4		

### CCL19 suppresses tumor angiogenesis in vitro

**Fig. 2:**
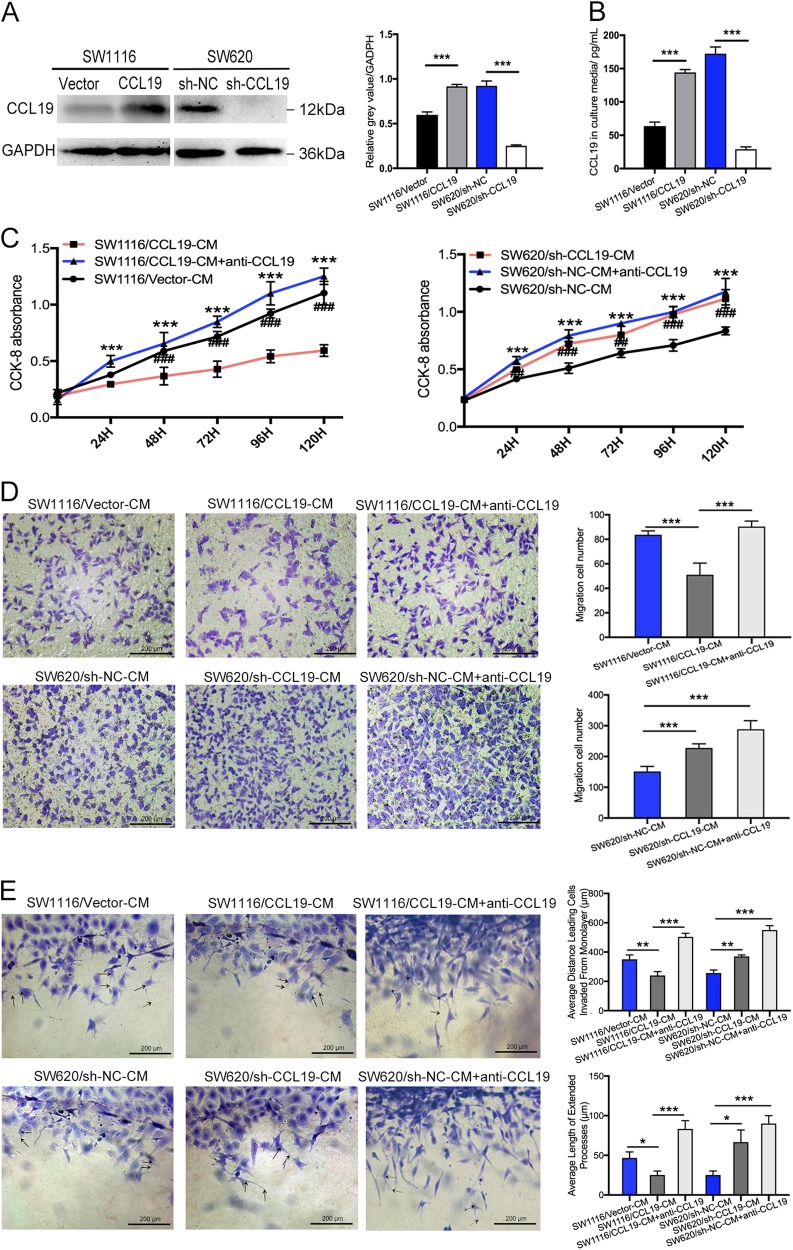
CCL19 inhibits proliferation, migration, and sprouting responses of HUVEC. **a** Verification of overexpression and knockdown of CCL19 in SW1116 and SW620 by immunoblot analysis. **b** The expression of CCL19 in culture media was detected by ELISA. **c** The proliferation ability was evaluated using CCK-8 assays in indicated HUVECs. **d** Representative images from the transwell migration assay in indicated HUVECs (magnification, 200×). **e** Images of 3D sprouting angiogenesis assay in indicated HUVECs (magnification, 200×). Average distance leading cells invaded from monolayer and average length of extended processes were used to evaluate the sprouting responses of HUVEC. Data represent the mean ± SD. **P < 0.05, **P < 0.01, ***P < 0.001

According to our previous results, we found that CCL19 was relatively low-expressed in SW1116 and high-expressed in SW620. Therefore, we generated stably overexpressing CCL19 in SW1116 cell line, as well as down-regulation of CCL19 in SW620, respectively. The effect of overexpression and knockdown was verified using western blot and ELISA (Fig. 2a, b). These cell lines were cultured for 24 h and their supernatant were collected and defined as SW1116/Vector-CM, SW1116/CCL19-CM, SW620/sh-NC-CM, SW620/sh-CCL19-CM, respectively. Next, we used these supernatants and anti-CCL19 neutralizing antibody to culture HUVEC or induce HUVEC migrate. The results of CCK-8 assay showed that SW1116/CCL19-CM can reduce the proliferation of HUVEC compared with SW1116/Vector-CM, and this effect can be blocked by anti-CCL19 neutralizing antibody. In contrast, SW620/sh-CCL19-CM can promote HUVEC proliferation compared with SW620/sh-NC-CM (Fig. 2c). We next investigated the function of these supernatant with high and low expression of CCL19 on HUVEC migration. The results showed that the SW1116/CCL19-CM, as well as the SW620/sh-CCL19-CM can either inhibit or promote the migration of HUVEC compared with the control group, which can be abrogated by anti-CCL19-neutralizing antibody (Fig. 2d). In 3D sprouting angiogenesis assay, SW1116/CCL19-CM can suppress HUVEC sprouting responses, whereas SW620/sh-CCL19-CM can promote HUVEC sprouting responses (Fig. 2e). And these effects can be blocked by anti-CCL19 neutralizing antibody. Similarly, the endothelial tube formation assay acquired the same results as 3D sprouting assay (Supplementary figure 4A).

In addition, recombinant human CCL19, recombinant human VEGF, and recombinant human HGF were used to stimulate HUVECs and their effect were observed by CCK-8 proliferation assay, transwell assay, and 3D sprouting assay. The results show that recombinant human CCL19 can inhibit the pro-angiogenesis effect of VEGF and HGF (Supplementary figure 2). Taken together, these results indicated that CCL19 can reduce the ability of HUVEC on proliferation, migration, and sprouting responses which may be able to suppress tumor angiogenesis in vivo.

### Tumor-derived CCL19 suppresses CRC angiogenesis in CCR7-dependent manner

**Fig. 3:**
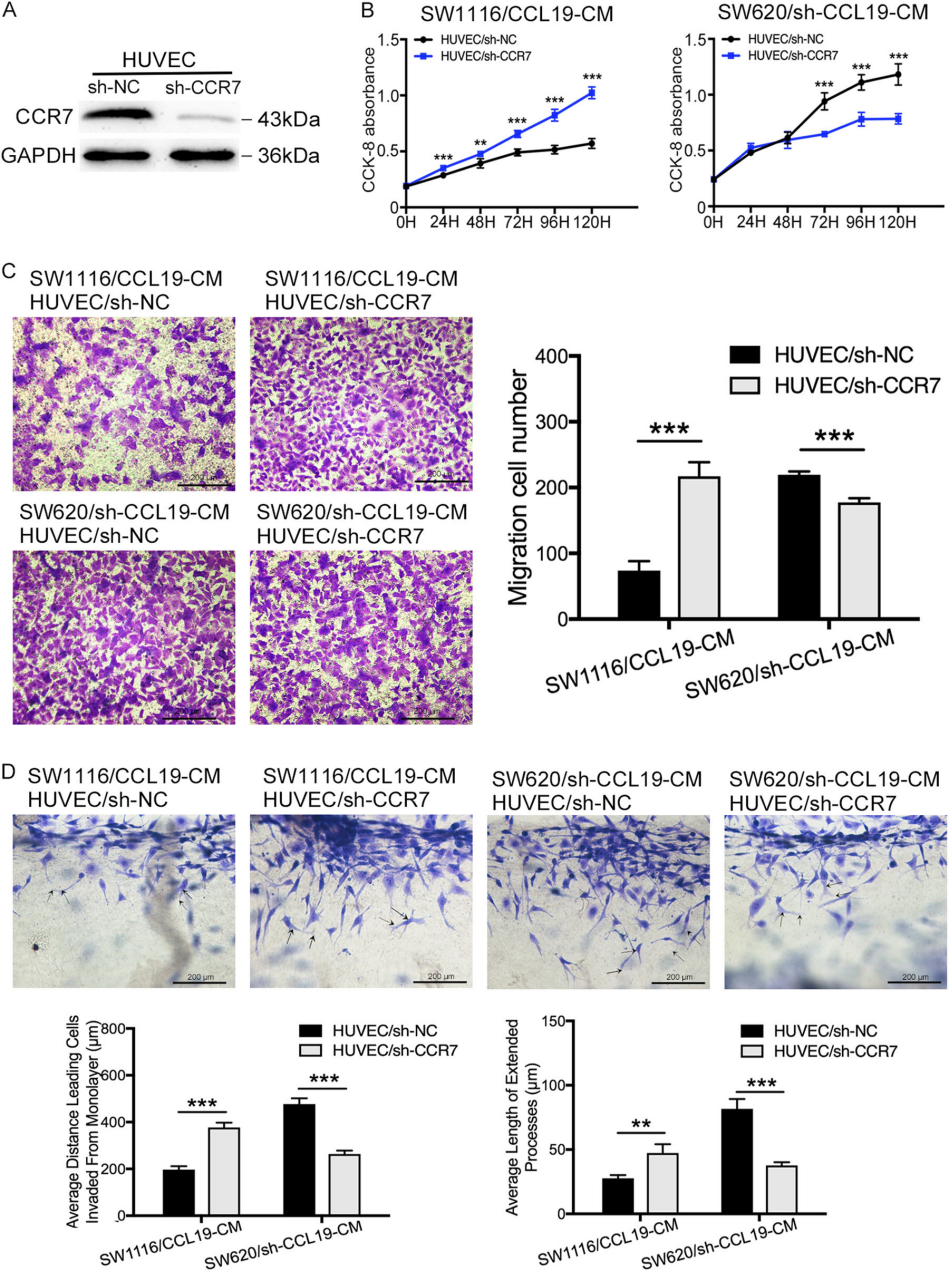
CCL19 inhibits proliferation, migration, and sprouting responses of HUVEC in a CCR7-dependent manner. **a** Verification of knockdown of CCR7 in HUVEC by immunoblot analysis. **b** The proliferation ability was evaluated using CCK-8 assays in indicated HUVECs. **c** Representative images from the transwell migration assay in indicated HUVECs (magnification, 200×). **d** Images of 3D sprouting angiogenesis assay in indicated HUVECs (magnification, 200×). Average distance leading cells invaded from monolayer and average length of extended processes were used to evaluate the sprouting responses of HUVEC. Data represent the mean ± SD. *P < 0.05, **P < 0.01, ***P < 0.001

We further investigated the role of CCR7 on CCL19-related tumor angiogenesis. We detected the expression of CCR7 in CRC cell lines and HUVEC, and found that CCR7 was significantly high-expressed in HUVEC compared with CRC cell lines (Supplementary figure 3A). So we hypothesized that the CCL19 secreted by CRC cells predominantly combined with CCR7 expressed by HUVEC and affect tumor angiogenesis. Knock-down of CCR7 was conducted in the HUVEC cells and expression was confirmed by immunoblotting (Fig. 3a). And then SW1116/CCL19-CM and SW620/sh-CCL19-CM were used to treat HUVEC/sh-CCR7 in CCK-8 proliferation assay, transwell assay, 3D sprouting angiogenesis assay, and endothelial tube formation assay to investigate if the process above was CCR7-dependent. Our results showed that the inhibition of CCR7 in HUVEC can neutralize the CCL19-related proliferation-suppressing behavior in HUVEC (Fig. 3b). In transwell assay, SW1116/CCL19-CM could not inhibit the migration of HUVEC/sh-CCR7 compared with HUVEC/sh-NC. Similarly, SW620/sh-CCL19-CM was not able to promote the migration of HUVEC/sh-CCR7 compared with HUVEC/sh-NC (Fig. 3c). The 3D sprouting angiogenesis assay showed that SW1116/CCL19-CM cannot suppress HUVEC/sh-CCR7 sprouting responses compared with HUVEC/sh-NC, whereas SW620/sh-CCL19-CM cannot promote HUVEC/sh-CCR7 sprouting responses compared with HUVEC/sh-NC (Fig. 3d). Similarly, the endothelial tube formation assay acquired the same results as 3D sprouting assay (Supplementary figure 4B). These results indicated that CCL19 suppressed CRC cell angiogenesis in a CCR7-dependent manner.

### CCL19 suppresses CRC angiogenesis through ERK/Elk-1/HIF-1α /VEGF-A pathway

**Fig. 4:**
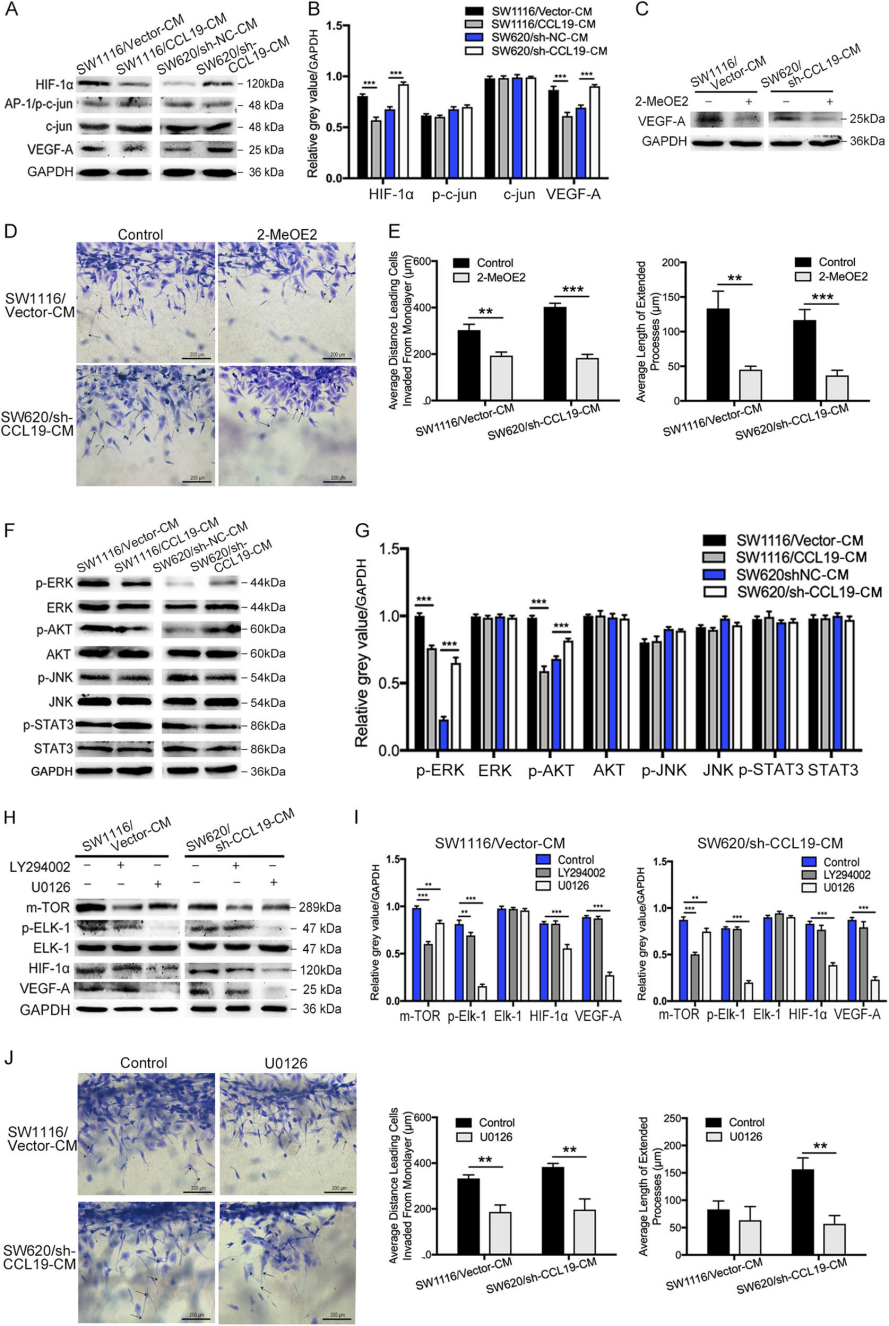
CCL19 suppresses angiogenesis through ERK/ELK-1/HIF-1α/VEGFA inhibition. **a** and **b** CCL19 inhibits the expression of HIF-1α and VEGF-A in HUVEC. No differences are observed in p-c-jun/AP-1 and c-jun. **c** 2-MeOE2 inhibits the expression of VEGF-A. **d** and **e** Results of 3D sprouting angiogenesis assays showed the inhibitory roles of 2-MeOE2 on indicated HUVEC (magnification, 200×). **f** and **g** CCL19 inhibits the phosphorylation of ERK and Akt in HUVEC. No differences are observed in STAT3 or JNK pathway. **h** and **i** Inhibition of the ERK pathway using U0126 downregulates p-Elk-1, HIF-1a, and VEGF-A expression. Inhibition of the AKT pathway using LY294002 is not able to change the expression of HIF-1a and VEGF-A. **j** Results of 3D sprouting angiogenesis assays showed the inhibitory roles of U0126 on indicated HUVEC (magnification, 200×). Average distance leading cells invaded from monolayer and average length of extended processes were used to evaluate the sprouting responses of HUVEC. Data represent the mean ± SD. *P < 0.05, **P < 0.01, ***P *<* 0.001

Several studies have demonstrated that HIF-1α, AP-1, and VEGF-A may be related to tumor microvessel formation and can promote tumor angiogenesis^17,18^. To analyze the mechanisms underlying CCL19-mediated CRC angiogenesis inhibition, we performed western blot to examine the expression of HIF-1α, AP-1, and VEGF-A. As mentioned above, HUVECs were cultured in tumor cell supernatant from SW1116/Vector, SW1116/CCL19, SW620/sh-NC, and SW620/sh-CCL19. So the protein extracted from HUVECs of these groups were defined as HUVEC-SW1116/Vector-CM, HUVEC-SW1116/CCL19-CM, HUVEC-SW620/sh-NC-CM, HUVEC-SW620/sh-CCL19-CM, repectively. Western blot results revealed that both of HIF-1α and VEGF-A were activated in HUVEC-SW1116/Vector-CM and HUVEC-SW620/ sh-CCL19-CM. and inhibited in HUVEC-SW1116/CCL19-CM and HUVEC-SW620/sh-NC-CM (Fig. 4a, b). Next, inhibitor of HIF-1α (2-MeOE2) were used to pre-treat HUVECs and the expression of VEGF-A was down-regulated by 2-MeOE2 (Fig. 4c), indicating that VEGF-A was induced through HIF-1α pathway. The 3D sprouting angiogenesis assay showed that the sprouting responses of 2-MeOE2 pre-treated HUVECs were obviously suppressed (Fig. 4d, e). In addition, the endothelial tube formation assay acquired the same results as 3D sprouting assay (Supplementary figure 4C). These results revealed that CCL19 suppress angiogenesis through HIF-1α/VEGF-A pathway.

Previous studies have shown that HIF-1α and VEGF-A can be induced through ERK1/2, PI3K/AKT, STAT3, and JNK pathways^19–22^. Thus, we also analyzed the expression of these proteins in tumor cell supernatant pre-treated HUVECs, and the results showed that low levels of pERK and pAKT were observed in HUVEC-SW1116/CCL19-CM compared with HUVEC-SW1116/Vector-CM, as well as high levels of pERK and pAKT were found in HUVEC-SW620/sh-CCL19-CM compared with HUVEC-SW620/sh-NC-CM (Fig. 4f, g). However, the expression of pSTAT3 and pJNK have no difference in these groups. Next, the inhibitors of ERK (U0126) and PI3K (LY294002) were used to investigate which pathways can mediate CCL19-related down-regulation of HIF-1α and VEGF-A. In addition, Elk-1 has been confirmed to be a downstream targets of ERK, and mTOR has been verified to be an effector of the AKT pathway^23,24^. So we examine the expression of pElk-1, Elk-1, mTOR, HIF-1α, VEGF-A in HUVECs, which were treated by tumor supernatant and inhibitors. The results indicated that HIF-1α and VEGF-A were induced through the activition of ERK/Elk-1 pathway instead of AKT/mTOR pathway (Fig. 4h, i). To further confirm the effect of this pathway on angiogenesis, HUVEC pre-treated by U0126 were used in 3D sprouting angiogenesis assay and showed that sprouting responses of U0126 pre-treated HUVEC were obviously suppressed (Fig. 4j). Similarly, the endothelial tube formation assay acquired the same results as 3D sprouting assay (Supplementary figure 4D). Taken together, these data demonstrate that CCL19 can inhibitor CRC angiogenesis via regulation of the ERK/Elk-1/HIF-1α/VEGF-A pathway.

### CCL19 inhibits ERK pathway through upregulating miR-206 and downregulating Met

**Fig. 5:**
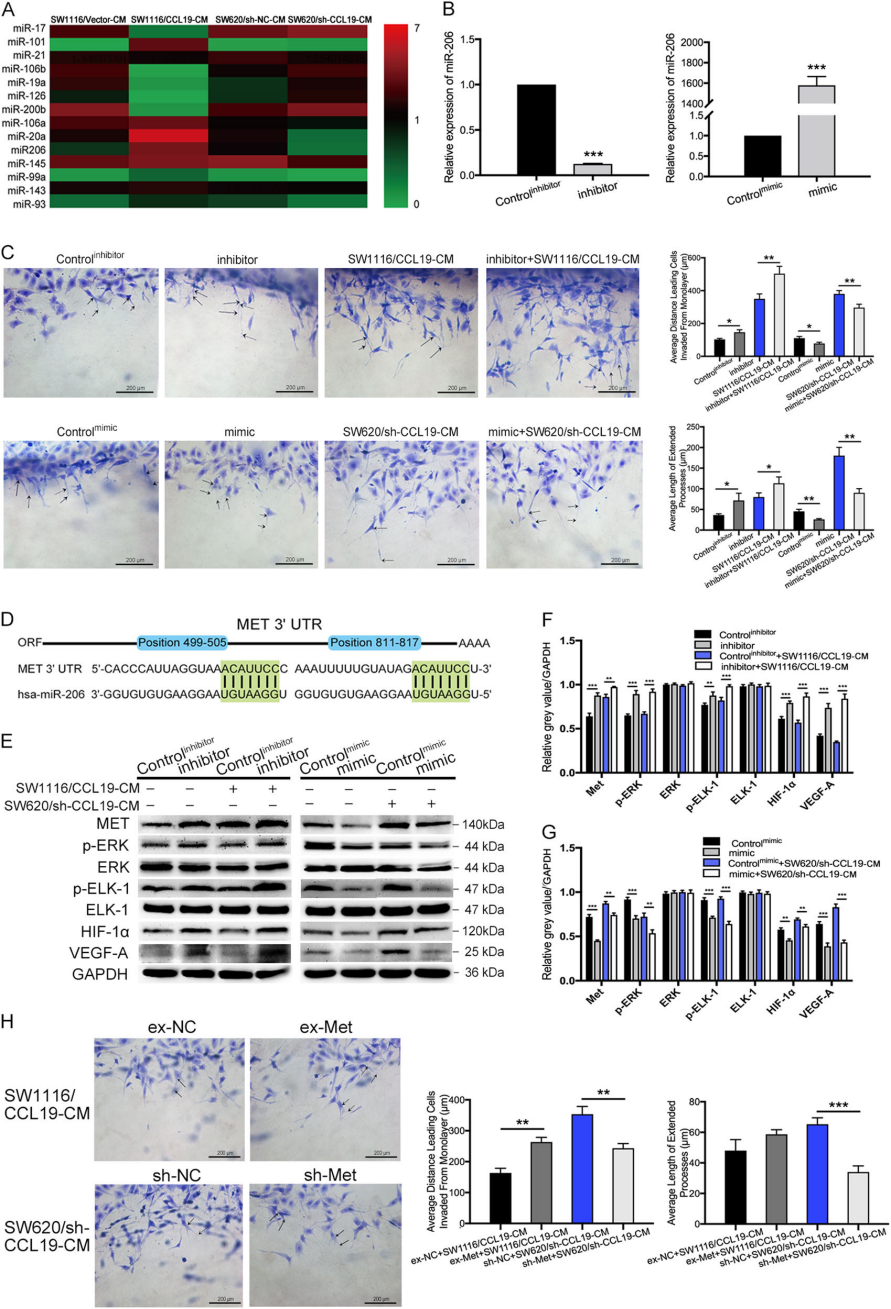
CCL19 inhibits ERK pathway through upregulating miR-206 and downregulating Met. **a** HUVEC cells were treated with tumor cell supernatants and then the expression of miRNAs were verified by qRT-PCR. Data are showed by 2-ΔΔCt. **b** HUVEC were transfected with miR-206 mimics and inhibitors, and transfection efficiency were verified by qRT-PCR. Data are shown by 2-ΔΔCt. **c** Images of 3D sprouting angiogenesis assay in indicated HUVECs (magnification, 200×). Average distance leading cells invaded from monolayer and average length of extended processes were significantly increased in miR-206 inhibitor groups and decreased in miR-206 mimic groups. **d** Graphic representation of the conserved miR-206-binding motifs within the 3′UTRs of MET. **e**, **g**, and **f** Met, ERK, p-ERK, Elk-1, p-Elk-1, HIF-1a, VEGF-A expressions were analyzed using western blot. Relative gray value represents the expression of the proteins relative to GAPDH. **h** Images of 3D sprouting angiogenesis assay in indicated HUVECs (magnification, 200×). Average distance leading cells invaded from monolayer and average length of extended processes were used to evaluate the sprouting responses of HUVEC. Data represent the mean ± SD. *P < 0.05, **P < 0.01, ***P *<* 0.001

It has been reported that chemokines can regulate the expression of many miRNAs^25^, so we detected the expression of CRC progression-related miRNAs in HUVECs treated by tumor cell supernatants using RT-PCR. The results showed that miR-20a and miR206 were up-regulated by CCL19, and miR-17, miR-19a, miR-200b were down-regulated by CCL19 (Fig. 5a and Supplementary figure 3B). Several studies reported that miR-206 can suppress the expression of VEGF-A and regulate tumor angiogenesis^26^, so miR-206 was selected for further investigation. Next, miR-206 mimic and inhibitor were used to explore the roles of miR-206 on CCL19-related tumor angiogenesis inhibition, and transfection efficiency were shown in Fig. 5b. Next, we performed 3D sprouting angiogenesis assay and the results demonstrated that miR-206 mimic reduced HUVEC sprouting responses, as well as miR-206 inhibitor promote HUVEC sprouting responses (Fig. 5c). Similarly, the endothelial tube formation assay acquired the same results as 3D sprouting assay (Supplementary figure 4E). We also predicted the potential binding sites for miR-206 within 3′UTRs of human genes by TargetScan (Fig. 5d). Among these potential targets of miR-206, Met had a high score and has been linked to tumor angiogenesis in previous reported articles^27^. It has been reported that the inactivation of Met can suppress tumor angiogenesis through inhibiting ERK signaling^28^. Thus, we performed western blot to analyze the expression of ERK/Elk-1/HIF-1α/VEGF-A signaling pathway proteins as mentioned above. As shown in Fig. 5e, high levels of Met, p-ERK, p-Elk-1, HIF-1α, and VEGF-A were observed in miR-206 inhibitor group compared with control group as well as low levels of Met, p-ERK, p-Elk-1, HIF-1α, and VEGF-A were observed in miR-206 mimic group compared with control group (Fig. 5e–g). To confirm the roles of Met in blunting CCL19‐induced anti-angiogenic effect, we ectopically over-expressed or knockdown Met in HUVECs, which were confirmed by Western blot (Supplementary figure 3C). 3D sprouting angiogenesis assay revealed that overexpression of Met promoted sprouting responses of HUVEC in SW1116/CCL19-CM, as well as knock-down of Met in HUVEC inhibited sprouting responses of HUVEC in SW620/sh-CCL19-CM (Fig. 5h). The results of western blot also showed that the expression of HIF-1α and VEGF-A in HUVEC were up-regulated by ex-Met and down-regulated by sh-Met (Supplementary figure 3D). Therefore, CCL19 suppressed angiogenesis by inhibiting Met. These results shown that CCL19-induced overexpression of miR-206 can suppress CRC angiogenesis by inhibiting Met/ERK/Elk-1/HIF-1α/ VEGF-A signaling.

### CCL19 reduces tumor angiogenesis in vivo

**Fig. 6:**
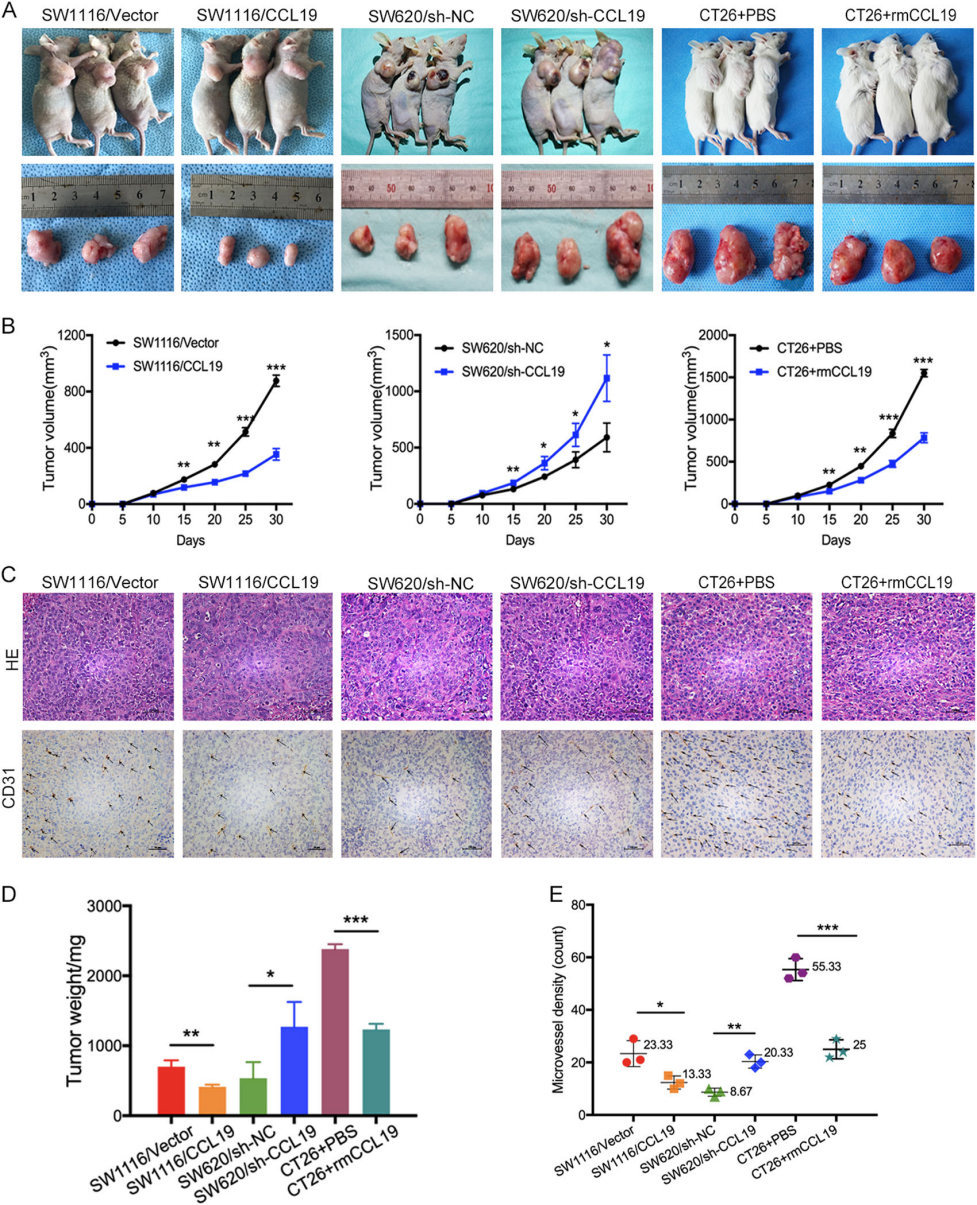
Effects of CCL19 on tumor growth and angiogenesis in vivo. **a** Images of xenografts in nude mice and Balb/c mice. **b** Tumor volume measured per week in different groups. **c** Images of IHC staining of CD31 of xenografts in nude mice and Balb/c mice. **d** Tumor weight in different groups (mg). **e** Number of microvessels in different groups (scale: 200 μm). Data are presented as the mean ± SD. *P < 0.05, **P < 0.01, ***P < 0.001

To determine whether CCL19 is involved in CRC angiogenesis in vivo, we constructed subcutaneous xenotransplanted tumor models. Results revealed that the average volume and weight of tumor nodules were increased in SW620/sh-CCL19 groups and decreased in SW1116/CCL19 groups and CT26 + rmCCL19 groups compared with control groups (Fig. 6a, b, d). In IHC staining analysis, more CD31-positive microvessel were observed in SW1116/Vector, SW620/sh-CCL19, and CT26 + PBS groups, as well as fewer CD31-positive microvessel were observed in SW1116/CCL19, SW620/sh-NC, and CT26 + rmCCL19 groups, respectively (Fig. 6c, e). These results indicate that CCL19 can reduce CRC angiogenesis in vivo.

To further verify the effect of CCL19 on suppressing angiogenesis expression was via Met and VEGF in vivo, SW620/sh-CCL19 xenotransplanted mice were treated with either a selective VEGF receptor tyrosine kinase inhibitor (axitinib), a MET inhibitor (crizotinib), or none. The results showed that the average volume and weight of tumor nodules were decreased and fewer CD31-positive microvessel were observed in axitinib-treated and crizotinib-treated mice (Supplementary figure 5). These results indicated that MET inhibitor and VEGF receptor inhibitor can reduce CRC angiogenesis in vivo.

## Discussion

Recent evidences show that chemokines and their receptors participated in tumor microenvironment and influence the progression of CRC^9^. It has been reported that CCL19 and CCR7 are able to inhibit metastasis in lung cancer and ovarian cancer^13,14^. Moreover, our previous studies revealed that low-expression of CCL19 significantly promoted CRC angiogenesis^16^. Consequently, this study mainly focuses on the potential molecular mechanisms and signal pathways underlying CCL19-related tumor angiogenesis.

**Fig. 7:**
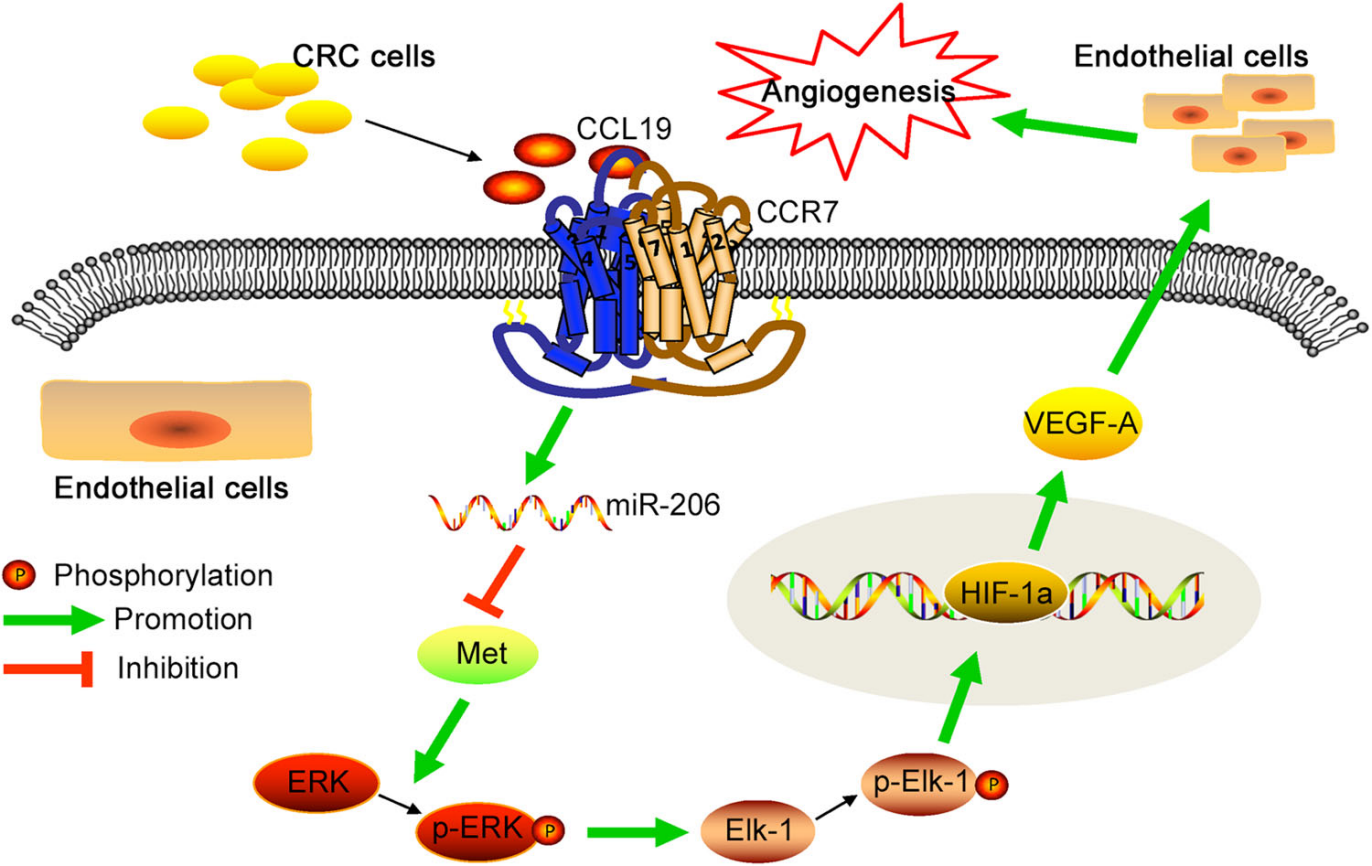
Schematic diagram shows that tumor-derived CCL19 combines with CCR7 expressed by endothelial cells and suppresses CRC angiogenesis. The function of CCL19 on angiogenesis is through inducing miR-206 thus inhibiting Met/ERK/Elk-1/HIF-1α/VEGF-A pathway, which can promote the proliferation, migration, and tube formation of ECs

As described above, we first detected tissue samples of CRC patients to identify the levels of CCL19 expression. Immunohistochemistry assay indicated that the expression of CCL19 was significantly down-regulated in CRC tissues compared with normal tissues. Next, we analyzed association between CCL19 expression and tumor MVD, then we found that CCL19 was significantly negativly correlated with tumor MVD. We also observed that CCR7 was primarily expressed in HUVEC instead of the CRC cell lines, as well as CRC cell lines have been reported to secrete CCL19. This result show that the function of CCL19 secreted from CRC cell may mainly depend on combining with CCR7 expressed on HUVEC. To further examine this process, we constructed CRC cell lines that overexpressed or down-expressed CCL19 as well as knocked down CCR7 expression in HUVEC. These stable transfected cells were used in both in vitro and in vivo experiments to explain the function of CCL19 on CRC angiogenesis inhibition. We also used mice CRC cell line CT26 and rmCCL19 to construct BALB/c subcutaneous xenotransplanted tumor models. The results indicated that up-regulated expression of CCL19 can decrease CRC angiogenesis in the nude mouse model, whereas in vitro experiments also showed that ectopic expression of CCL19 can inhibit the proliferation, migration, and tubules formation ability of HUVEC (Fig. 7).

Angiogenesis is a fundamental step in tumorigenesis and metastasis via synthesis of new tumor microvessel^29^. This process is closely related to some angiogenesis factors, such as VEGF-A, HIF-1α, and AP-1^30,31^. Herein, the expression levels of VEGF-A and HIF-1α were decreased under condition of CCL19 overexpression, while knockdown of CCL19 increased the expression of VEGF-A and HIF-1α in HUVEC. These data provide that CCL19 inhibits CRC angiogenesis might be through down-regulation of VEGF-A and HIF-1α. We then explored the potential signal pathways in down-regulation of VEGF-A and HIF-1α, and the results demonstrated that both ERK and PI3K/Akt pathways probably involved in this process. Small chemical inhibitors against ERK (U0126) and AKT (LY294002) were used, and the results investigated that the inhibition of the ERK pathway rather than AKT pathway leads to the alteration of angiogenesis factors. It has been reported that Elk-1 is one of the ERK downstream transcription factors^32^. In this study, we demonstrated that CCL19 can inhibit the ERK/Elk-1 pathway, which is accompanied by the suppression of HIF-1α and VEGF-A.

MicroRNAs (miRNAs) are a type of noncoding RNAs which can silence the target gene by binding to the 3′-untranslated regions (3′-UTR) of specific mRNAs^33^. Increasing evidences indicate that miRNAs have been involved in tumorigenesis and progression^34^. For example, MiR-17-5p can enhance pancreatic cancer proliferation via disruption of RBL2/E2F4-repressing complexes^35^. In the recent studies, many chemokines can be regulators of miRNAs^25,36^. To identify specific CCL19-regulated miRNAs alterations, we detected the expression of CRC progression-related miRNAs in HUVECs treated by tumor cell supernatants using RT-PCR. From these data, two miRNAs (miR-20a and miR206) were up-regulated and three miRNAs (miR-17, miR-19a, miR-200b) were down-regulated in the CCL19 overexpression tumor cell supernatants treated HUVECs. Specially, miR-206 has been observed being down-expressed in various types of cancers, such as lung cancer^37^, breast cancer^38^, gastric cancer^39^, and hepatocellular cancer^40^, and also be able to suppress the expression of VEGF-A and regulate the tumor angiogenesis^26^. It has been confirmed that miR-206/Met can inhibit migration and tube formation of HUVEC in vitro and increased MVD in vivo nude mice^41^. Similarity, our study find that ectopic expression of CCL19 is significantly associated with high expression of miR-206, leading to suppression of CRC angiogenesis, and Met might be the downstream target of miR-206. However, miR-206 inhibitors reverse this process. In conclusion, CCL19 plays an important role in angiogenesis inhibition by activating miR-206 expression and subsequently inhibiting Met/ERK/Elk-1/HIF-1α/VEGF-A signaling axis in CRC microenvironment.

Tumor angiogenesis is important for tumorigenesis and progression^42^. In CRC, tumor metastasis and angiogenesis are the major cause of death and poor prognosis of CRC patients^37^. Nowadays, drugs against angiogenesis, such as bevacizumab have been put into clinical treatment of metastasis CRC, indicating that anti-angiogenic therapy has broad prospects^43^. The proliferation, migration, and tube formation of endothelial cells (ECs) are important for tumor angiogenesis, so we used HUVECs to conduct our in vitro experiments in this study. Although the function of CCL19/CCR7 on tumor angiogenesis has been confirmed in our study, it is difficult to translate the results obtained in experiments to human disease treatment, and the internal mechanisms of tumor angiogenesis still require to be further studied.

## Conclusion

In conclusion, this study reveal that CCL19 is low-expressed in CRC tissues, which links to highly tumor MVD. CCL19 suppresses angiogenesis in CRC via promoting miR-206 thus inhibiting Met/ERK/Elk-1/HIF-1α/VEGF-A pathway in a CCR7-dependent pattern. These findings support that activation of the CCL19/CCR7 signaling pathway may be a promising option of anti-vascular treatment for CRC. However, we discussed the anti-angiogenic effect of CCL19 using HUVEC only in our article. Therefore, there are still some limits of our research. The effect of CCL19 on tumor angiogenesis remains to be studied further.

## Methods

### Patients and specimens

The collection of specimens from 78 patients were performed after obtainning the informed consent from the Biomedical Ethics Committee of Ruijin Hospital. All these patients were diagnosed as CRC pathologically and accepted laparoscopic surgery in Minimally Invasive Surgery Centre, Ruijin Hospital, Shanghai Jiaotong University. Patients who accepted preoperative treatment such as radiation or chemotherapy were excluded from our study. These tissues were embedded with paraffin and made to tissue microarray (TMA) by Shanghai Outdo Biotech Company (Shanghai, China), as previously described^44^.

### Immunohistochemical (IHC) analysis and microvessel counting

The IHC staining of TMA was performed according to the manufacturer’s protocol (Immunostain SP kit, DakoCytomation, USA). Antibodies of IHC analysis included anti-CCL19 (AF361, R&D) and anti-CD31 (sc-376764, Santa Cruz Biotechnology) antibody. The IHC staining scores of CCL19 in tumor tissues were determined by two independent pathologists according to semi-quantitative immunoreactivity scoring (IRS) system^45^. Intensity of immunostaining was scored as 0 (no immunostaining), 1 (weak immunostaining), 2 (moderate immunostaining), and 3 (strong immunostaining). The percentage of IHC cells scoring was documented as 0 (none), 1 (<10%), 2 (10–50%), 3 (51–80%), and 4 (>80%)^46^. The intensity of immunostaining score and the percentage of IHC cells score were multiplied to generate IRS ranging from 0 to 12 for each tumor. The microvessel counting was done according to the method proposed by Weidner et al.^47^. Briefly, the most intense area of tumor microvessel was identified by light microscopy. Next, we performed individual microvascular counts of these most intense area in a 200× field. Any CD31-positive endothelial cell or endothelial cell cluster which clearly separated from adjacent cells was considered as a single microvascular that can be counted. The highest number of microvessels in any 200× field was defined as MVD.

### Cell lines and reagents

The human CRC cell lines used in our study were purchased from the American Type Culture Collection (ATCC, USA). HUVEC and CT26 were purchased from Shanghai Institutes for Biological Sciences, Chinese Academy of Sciences. All these cells were cultured in RPMI-1640 medium with 10% fetal bovine serum (FBS), penicillin (10^7^ U/l), and streptomycin (10 mg/l) and incubated at 37 °C and 5% CO_2_. Recombinant human HGF protein, recombinant human CCL19/MIP-3 beta protein, recombinant human VEGF 165 protein were purchased from R&D systems. Axitinib (VEGF receptor tyrosine kinase inhibitor) and crizotinib (MET inhibitor) were purchased from Selleckchem (Houston, USA) and used in accordance with manufacturer’s instructions.

### Lentivirus vectors and shRNA transfection

LV5-EF1a-GFP/Puro-CCL19 and LV3-pGLV-h1-GFP/Puro-shCC19 lentiviral vector were constructed by the Shanghai GenePharma Corporation (Shanghai, China), and shRNA plasmids targeting CCR7 mRNA were purchased from Obio Technology (Shanghai, China). Met shRNA expression vector, Met overexpression vector, and the control vector were constructed by the Shanghai GenePharma Corporation (Shanghai, China). The sequences of shCCL19, sh-Met, and shCCR7 are shown in Supplementary Table 1. Lentivirus transfection and plasmids transfection were performed as described previously^8^. Antibiotics puromycin was used to select stable transfected cells. The overexpression and interfering effects of these vectors/shRNAs were evaluated by immunoblot.

### RNA extraction and quantitative real-time PCR (qRT-PCR)

Total RNA was extracted by TRIzol (Invitrogen, USA). The quality and concentration of RNA were evaluated by a spectrophotometer (Bio-Rad, USA). cDNA was synthesized by miRNA First Strand cDNA Synthesis Kit (Sangon Biotech, China). The miRNAs Quantitation PCR Kit (Sangon Biotech, China) was used for the qPCR analysis of miRNA expression. The expression level of miRNAs was determined by qRT-PCR and normalized using U6 small nuclear RNA (Sangon Biotech, China) by the 2^−ΔCT^ method. The relative expression ratio of miRNAs in each group was calculated by the 2^−ΔΔCT^ method.

### miRNA transient transfection

The miR-206 mimics, negative control (NC), miR-206 inhibitor, and inhibitor NC were synthesized by GenePharma (Shanghai, China). Transfection was performed by Lipofectamine 3000 Reagent (Invitrogen, Carlsbad, CA, USA) according to the manufacturer’s protocol. Transfection efficiency was determined by qRT-PCR.

### 3D sprouting angiogenesis assay

3D sprouting angiogenesis assay were performed as described previously^48^. 3D collagen matrices were prepared at a final concentration of 2.5 mg/ml containing Sphingosine 1-phosphate (S1P, Sigma-Aldrich). A total of 100 μl of collagen was added per well of six-well plate (Corning), and allowed to polymerize at 37 °C with 5% CO_2_ for 1 h. HUVECs (80,000) were resuspend in a 200 μl M199 containing RSII, VEGF, FGF-2, and ascorbate, and added around the polymerized collagen matrices. After 1 h, an additional 800 μl of cell media were added (final concentration: 1:250 RSII, 40 ng/ml VEGF, 40 ng/ml bFGF, 50 μg/ml ascorbate). HUVECs were allowed to invade for 24 h at 37 °C with 5% CO_2_ before fixing in 3% glutaraldehyde (Sigma-Aldrich) in PBS and staining with 0.1% toluidine blue (Sigma-Aldrich) in 30% methanol. Finally, collagen matrices were photographed by microscopy and evaluated as described by Colette A. Abbey^49^.

### Endothelial tube formation assay

Tumor cells supernatant was collected from CCL19 overexpression and knockdown stable cells, and HUVECs were cultured with these tumor cells supernatant for 24 h before performing endothelial tube formation assay. Matrigel (BD Biosciences) was uniformly coated on each well of 96-well plate at 50 μl/well and incubated for 30 min at 37 °C, 5% CO_2_ for polymerize. Pre-treated HUVEC were plated on the matrigel at concentration 2 × 10^4^ cells/well and incubated for 3 h at 37 °C, 5% CO_2_. Tubules were photographed by microscopy and evaluated by ImageJ software.

### CCK-8 cell proliferation assay

HUVEC were pre-treated as reported in endothelial tube formation assay, and were seeded in 96-well plate at concentration of 2 × 10^3^ cells/well within tumor cells supernatant in 37 °C, 5% CO_2_ incubator. The viability of cells was determined at every 24 h. Before the test, 10 μl CCK-8 (Dojindo Molecular Technologies, Kumamoto, Japan) was added into each well and incubated at 37 °C for 2 h, and the OD values were detected using spectrophotometer (BioTek, Winooski, USA).

### Cell migration assay

Transwell chamber was used (8 μm for 24-well plate; Corning Costar, NY, USA) to perform cell migration assay. 200 μl serum-free medium containing 5 × 10^4^ HUVEC was added into the upper transwell chamber while 800 μl tumor cells supernatant was added into the lower chamber. After 18 h incubation, HUVEC cells were fixed with methanol and stained with 0.5% crystal violet for 30 min. Finally, HUVEC on the lower side of chamber membrane were counted and photographed by microscope.

### Immunoblotting

Western blot analysis was performed as previously described^50^. 100 μg of protein was separated by 12.5% SDS–PAGE gel and transferred to PVDF membranes. The membranes were blocked with 5% bovine serum albumin (BSA) for 2 h and then were incubated with primary antibodies at 4 °C overnight. The primary antibodies included CCL19 (R&D), VEGF-A (Abcam), HIF-1α (Abcam), ERK (Cell Signaling), p-ERK (Cell Signaling), Akt (Cell Signaling), p-Akt (Cell Signaling), Elk-1 (Cell Signaling), p-Elk-1 (Cell Signaling), JNK (Santa Cruz), p-JNK (Santa Cruz), STAT3 (Santa Cruz), p-STAT3 (Santa Cruz), c-jun (Santa Cruz), p-c-jun (Santa Cruz), and Met (Santa Cruz). Horseradish peroxidase-conjugated secondary antibodies were used and the protein bands were visualized by an enhanced chemiluminescence detection system (Amersham Bioscience, Piscataway, NJ, USA) according to the manufacturer’s protocol.

### Mice tumorigenesis

Stable transfected CRC cells (1 × 10^6^ cells) were subcutaneously injected into 4-week-old male nude mice (Institute of Zoology, China Academy of Sciences), as well as CT26 cells were subcutaneously injected into BALB/c mice. In addition, we injected 0.2 μg recombinant mouse CCL19 (rmCCL19, R&D) in the tumor site of BALB/c mice twice a week^16^. Treatments began approximately 10 days later, when tumors were detectable by palpation. Crizotinib (25 mg/kg, 1×/day) and axitinib (30 mg/kg, 2×/day) were administered by oral gavage during 20 days. Tumor nodules were measured every 5 days, and the mice were euthanized 4 weeks after injection. Tumors were weighed and fixed by formalin. Tumors IHC staining and microvessel counting were as mentioned before. All steps were performed according to the Guide for the Care and Use Laboratory Animals of Ruijin Hospital, Shanghai Jiaotong University School of Medicine.

### Statistics

All of the statistical analysis were performed by SPSS 20.0 software or R software 3.1.2 (R Core Team). The Pearson *χ*^2^-test and Fisher’s exact probability method were used to analyze the relationship between CCL19 and clinical features. Quantitative variables were analyzed by Student's *t*-test. The correlations between CCL19 and MVD were analyzed by Pearson correlation coefficient. Data were shown in mean ± SD. All experiments were performed in triplicate. A two-tailed value of *P* < 0.05 was considered statistically significant.

### Availability of data and materials

The datasets supporting the conclusions of this article are included within the article and its additional files.

## Electronic supplementary material

Supplementary Figure 1

Supplementary Figure 2

Supplementary Figure 3

Supplementary Figure 4

Supplementary Figure 5

Supplementary Table 1

Supplementary figure legends
