# Supplementary figures and images for: CCL19 suppresses angiogenesis through promoting miR-206 and inhibiting Met/ERK/Elk-1/HIF-1α/VEGF-A pathway in colorectal cancer
Source: Cell Death Dis. 2018 Sep 24;9(10):974. doi: 10.1038/s41419-018-1010-2 (PMC6155262; doi:10.1038/s41419-018-1010-2)

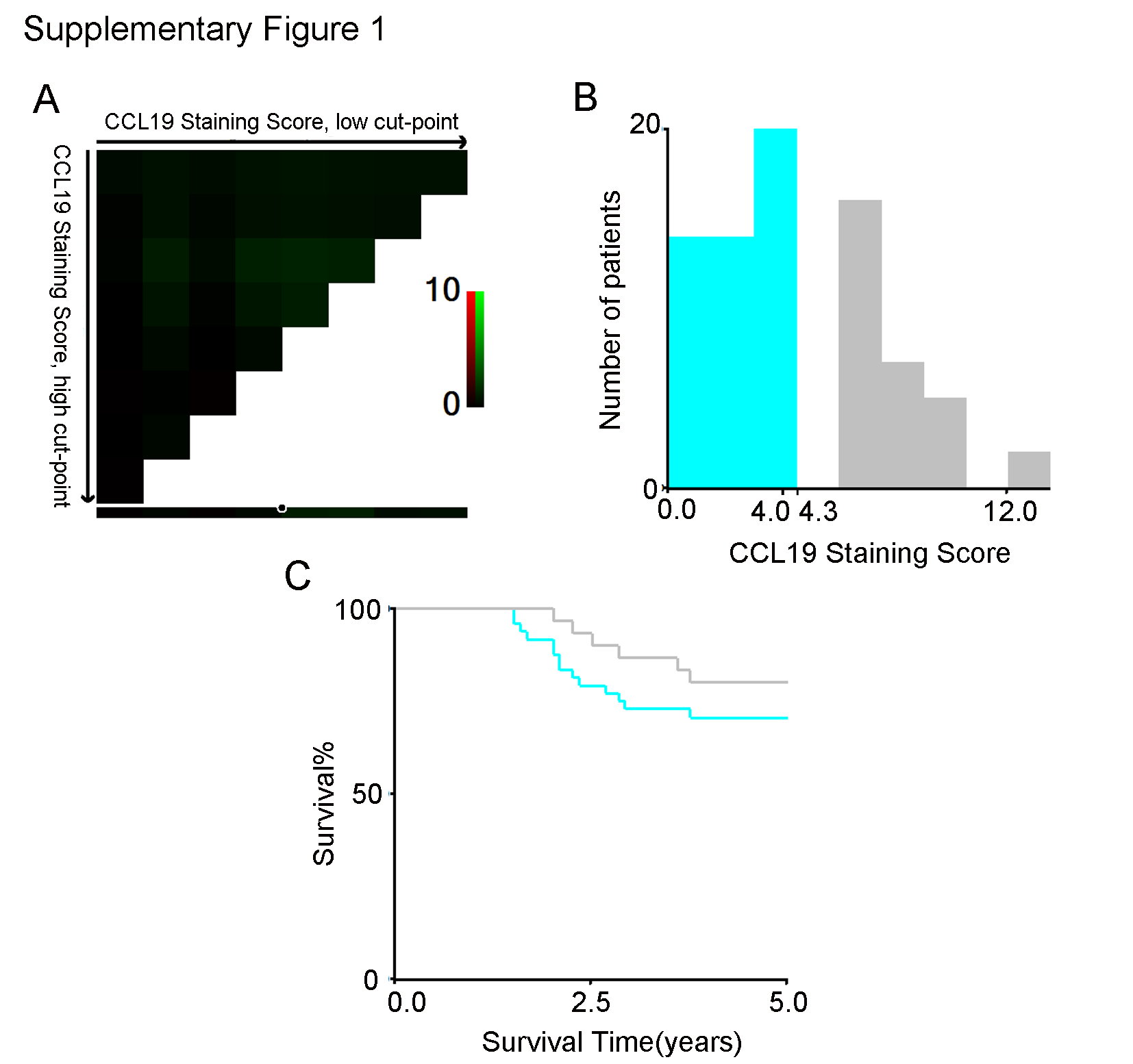

Supplement: Supplementary file 1 — Supplementary Figure 1 [file 41419_2018_1010_MOESM1_ESM.tif]

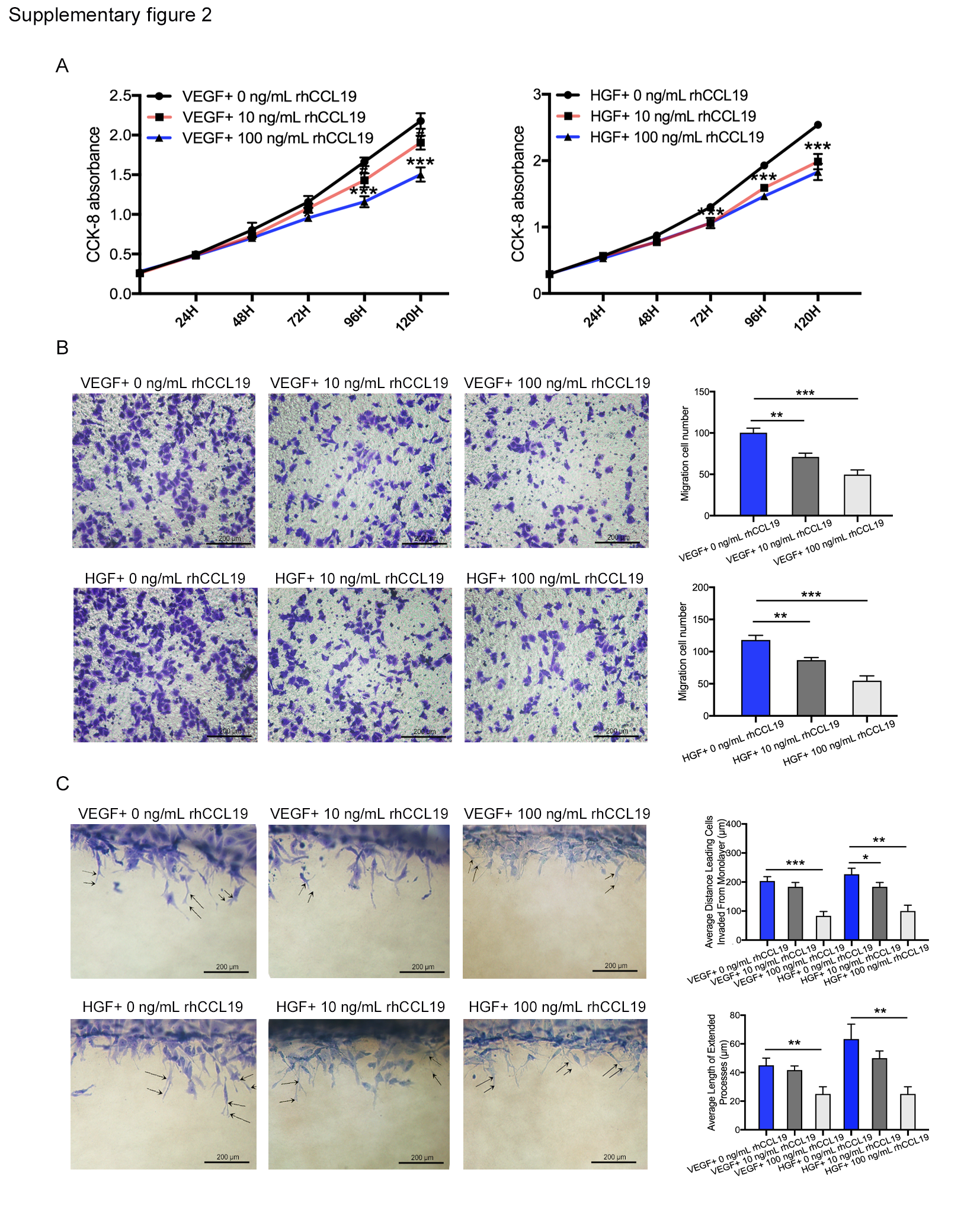

Supplement: Supplementary file 2 — Supplementary Figure 2 [file 41419_2018_1010_MOESM2_ESM.tif]

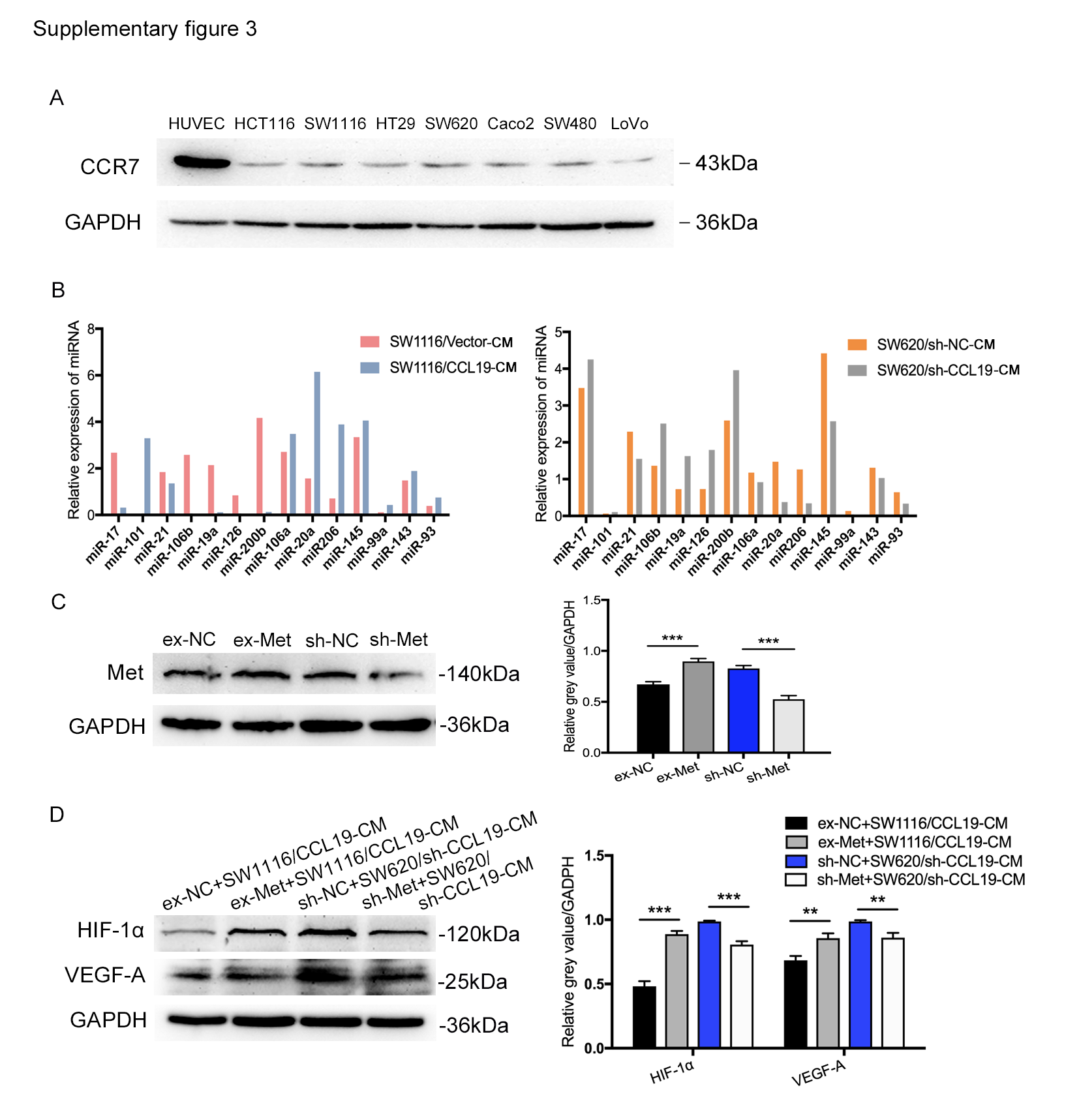

Supplement: Supplementary file 3 — Supplementary Figure 3 [file 41419_2018_1010_MOESM3_ESM.tif]

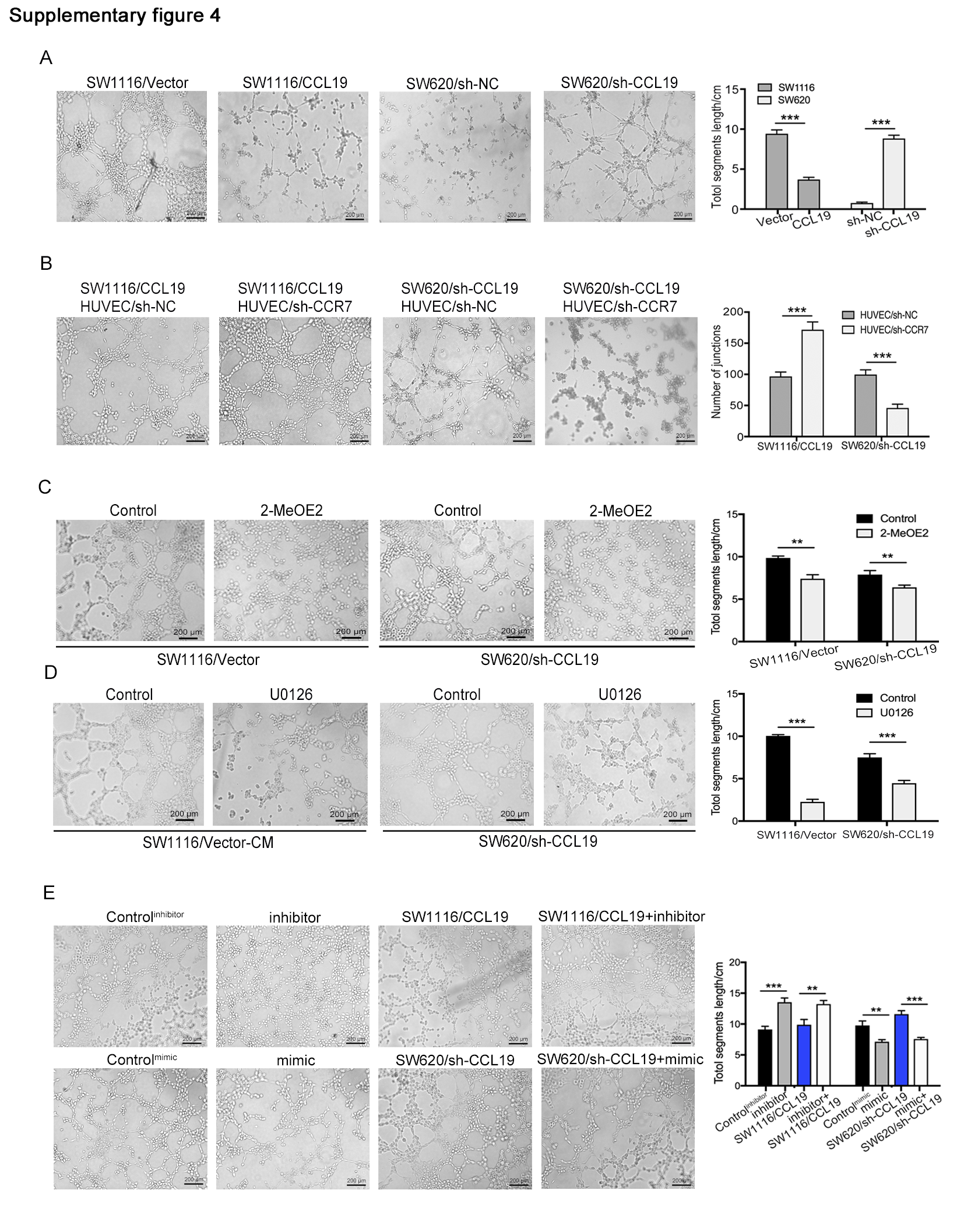

Supplement: Supplementary file 4 — Supplementary Figure 4 [file 41419_2018_1010_MOESM4_ESM.tif]

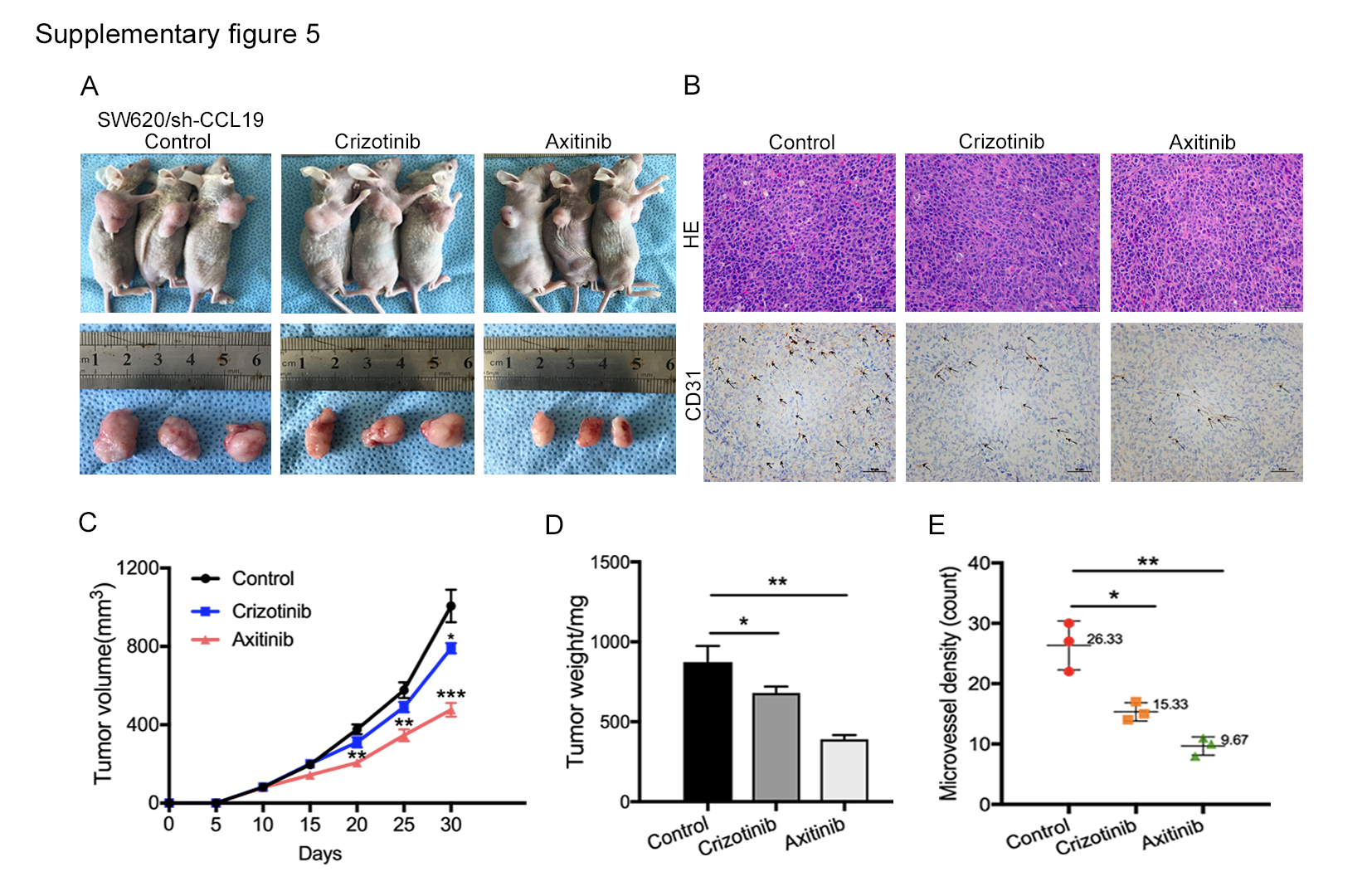

Supplement: Supplementary file 5 — Supplementary Figure 5 [file 41419_2018_1010_MOESM5_ESM.tif]
