# Supplementary material for: CCL19 suppresses angiogenesis through promoting miR-206 and inhibiting Met/ERK/Elk-1/HIF-1α/VEGF-A pathway in colorectal cancer
Source: Cell Death Dis. 2018 Sep 24;9(10):974. doi: 10.1038/s41419-018-1010-2 (PMC6155262; doi:10.1038/s41419-018-1010-2)
Supplement: Supplementary file 6 — Supplementary Table 1 [file 41419_2018_1010_MOESM6_ESM.docx]

**Supplementary Table 1 shRNA Sequences**

| shRNA | Stem Sequence |
| --- | --- |
| CCL19-LV3-shRNA#1  **CCL19-LV3-shRNA#2**  CCL19-LV3-shRNA#3 | CACTACCTTCTCATCAAGGAT  **CCCTGGGTACATCGTGAGGAA**  GCCCTGGGTAGAACGCATCAT |
| CCR7-LV3-shRNA#1  **CCR7-LV3-shRNA#2**  CCR7-LV3-shRNA#3 | TACACTTTGTTCGAGTCTTTG  **GATGAGGTCACGGACGATTAC**  GCTGGTCGTGTTGACCTATAT |
| **Met-LV3-shRNA#1**  Met-LV3-shRNA#2  Met-LV3-shRNA#3 | **TCAGAACCAGAGGCTTGGT**  GAAGTGATTGTGGAGCATA  CTGCTTTAATAGGACACTT |

***Bold fonts represent the most effective sequence**
