# Supplementary material for: CCL19 suppresses angiogenesis through promoting miR-206 and inhibiting Met/ERK/Elk-1/HIF-1α/VEGF-A pathway in colorectal cancer
Source: Cell Death Dis. 2018 Sep 24;9(10):974. doi: 10.1038/s41419-018-1010-2 (PMC6155262; doi:10.1038/s41419-018-1010-2)
Supplement: Supplementary file 7 — Supplementary figure legends [file 41419_2018_1010_MOESM7_ESM.docx]

**Supplementary figure legends**

**Supplementary Figure 1**

X-tile analysis of survival data in CRC patients reveals a continuous distribution based on CCL19 staining score. The plot shows the χ2 log-rank values produced when dividing the cohort with one cut-point, producing high, and low subsets. The X-axis represents all potential cut-points from low to high (left to right) that defines a low subset, whereas the Y-axis represents cut-points from high to low (top to bottom), that defines a high subset. Red coloration of cut-point indicates an inverse correlation with survival, whereas green coloration represents direct associations (A). The optimal cut-point occurs at the brightest pixel (red). The cut-point highlighted by the white circle in A is shown on a histogram of the entire cohort (B), and a Kaplan-Meier plot (C, low subset grey, high subset light green). (TIF 4341 kb)

**Supplementary Figure 2**

CCL19 inhibits proliferation, migration and sprouting responses of HUVEC which treated by using VEGF and HGF. (A) The proliferation ability was evaluated using CCK-8 assays in indicated HUVECs. (B) Representative images from the transwell migration assay in indicated HUVECs (Magnification, 200×). (C) Images of 3D sprouting angiogenesis assay in indicated HUVECs (Magnification, 200×). Average Distance Leading Cells Invaded From Monolayer and Average Length of Extended Processes were used to evaluate the sprouting responses of HUVEC. Data represent the mean ± SD. **P < 0.05, **P < 0.01, ***P < 0.001.

**Supplementary Figure 3**

(A) The expression of CCR7 were determined by western blot analysis. CCR7 was significantly high expressed in HUVEC. (B) HUVEC cells were treated with tumor cell supernatants and then the expression of miRNAs were verified by qRT-PCR. Data are showed by 2-ΔΔCt. (C) Verification of overexpression and knockdown of Met in HUVEC by western blot. (D) HIF-1a, and VEGF-A expressions were analyzed using western blot. Relative grey value represents the expression of the proteins relative to GAPDH. Data represent the mean ± SD. **P < 0.05, **P < 0.01, ***P < 0.001.

**Supplementary Figure 4**

Images of tube formation assay in indicated HUVECs (Magnification, 200×). Total segments length were used to evaluate the tube formation ability of HUVEC. Data represent the mean ± SD. **P < 0.05, **P < 0.01, ***P < 0.001.

**Supplementary Figure 5**

Effects of Met and VEGF on tumor growth and angiogenesis in vivo. (A) Images of xenografts in nude mice. (B) Images of IHC staining of CD31 of xenografts in nude mice. (C) Tumor volume measured per week in different groups. (D) Tumor weight in different groups (mg). (E) Number of microvessels in different groups (Scale: 200 μm). Data are presented as the mean ± SD. *P < 0.05, **P < 0.01, ***P < 0.001.

**Supplementary Table 1**

The sequences of shCCL19 and shCCR7 are shown in Supplementary Table 1. Bold fonts represent the most effective sequence.
